# A Latent Profile Analysis of Chinese Physicians' Workload Tethered to Paperwork During Outpatient Encounters

**DOI:** 10.3389/fpubh.2022.854772

**Published:** 2022-04-25

**Authors:** Dehe Li, Yinhuan Hu, Sha Liu, Chuntao Lu, Jiayi Li, Jinghan Zhou, Yeyan Zhang, Shaoyu Lu

**Affiliations:** ^1^School of Medicine and Health Management, Tongji Medical College, Huazhong University of Science and Technology, Wuhan, China; ^2^Jingmen No. 2 People's Hospital, Jingmen, China

**Keywords:** physician workload, subgroups, non-physician-patient communication work tasks, paperwork, outpatient encounter, LPA, clerical burden, China

## Abstract

**Background:**

Physician dissatisfaction with more time spent on related paperwork but less time available for direct interaction with patients is increasing internationally. Increased physician workload resulting from paperwork might negatively affect their interaction with patients and increase the risk for burnout. This study aimed to investigate the level of physician workload tethered to paperwork during outpatient encounters and explore its latent workload subgroups among Chinese physicians.

**Methods:**

A cross-sectional survey was conducted via online questionnaire primarily in 24 hospitals in 6 provinces in Eastern, Central, and Western China from November 2020 to February 2021. The Chinese physician mental workload scale developed by our research team was used for assessment of physician workload tethered to paperwork. Physicians were categorized into different subgroups of workload via latent profile analysis. Multinomial logistic regression was subsequently performed to examine how demographic variables differ among physicians belonging to different subgroups.

**Results:**

A total of 1,934 valid questionnaires were received. Chinese physicians reported medium levels of workload while performing non-physician-patient communication work tasks characterized by paperwork during outpatient encounters. Four latent workload subgroups were identified: “low workload group” (8.8%), “medium workload group” (34.0%), “high workload group” (42.1%) and “very high workload group” (15.1%). Compared with the other latent workload subgroups, physicians belonging to the “very high workload group” were more likely to be younger, married, those who had worse health status, lower educational levels and lower average monthly incomes, those who worked more years in the current institution, more hours per week and longer outpatient hours per week, and those who worked in public general hospitals, tertiary B hospitals and Obstetrics and Gynecology, and saw more than 50 outpatients per day, with more time spent on per patient.

**Conclusions:**

There exit four latent workload subgroups among Chinese physicians tethered to paperwork during outpatient encounters along with great individual variations among these subgroups. The characteristics of the latent “very high workload group” can help permit more targeted guidance for developing interventions with optimized human resource allocation to, in turn, increase the time available for direct interaction with patients, thereby resulting in improved quality of physician-patient interactions and decreased risk for physician burnout.

## Introduction

With the increasing aging population with chronic and age-related diseases, along with its subsequently increasing health care requirements worldwide (1, 2), alarming increasing trends of physician workload have received much attention from health care providers and decision makers, as well as researchers in recent years (3–6). The well-known physician shortage issue in China [2.04 practicing physicians per 1,000 residents in 2017 (7), compared with the international average of 3.5 (8)] could further contribute to a much heavier workload for Chinese physicians. Heavy workload in physicians can contribute to an increased risk for burnout (9, 10), negatively affect their health (11–13), and further lead to an inferior quality of patient care (3, 14), negative patient satisfaction (15) and even medical errors (10), eventually endangering patient safety (16).

Workload is a multidimensional and multifaceted construct (17), comprising objective workload that is simply reflected by the quantity of work tasks, and mental workload that reflects the mental strains resulting from performing a work task under a specific environmental or operational condition as well as the capability of the human operator to respond to those demands (11). Compared to the objective workload, mental workload not only reflects different aspects of a human operator's workload, but also explains the relation between the nature of a work task and the characteristics of the operator (18, 19); and currently, the European Pact for Mental Health and Welfare is devoted to conducting the mental workload assessments to promote physical and mental well-being (11). To date, various methods for quantifying mental workload have been developed, mainly including the following three large groups: subjective evaluations through rating scales, task performance measures, and physiological measures (e.g., heart rate, galvanic skin resistance, and breathing rate) (20, 21); and the NASA-Task Load Index (NASA-TLX) scale provides a well-validated and widely-used tool for measuring or diagnosing subjective mental workload (22, 23), and has been used to quantify perceived workload of healthcare workers in various healthcare settings (24).

In China, heavy workload in physicians is a major problem for the current health care system (10), seriously threatening their health. Not surprisingly, the issue of overwhelming workload for physicians has attracted great public concern because of continuous cases involving young and middle-aged physicians' sudden death in recent years. And the current COVID-19 epidemic has further contributed to an increased work burden for Chinese medical workers including physicians than before, especially in the center of the breakout of COVID-19 epidemic, where they suffered significant mental health problems during the COVID-19 outbreak (25–27). However, Chinese patients still tend to go to high-level hospitals even for mild symptoms owing to their lack of confidence in the quality of health care provided in primary hospitals (10, 28); and with the growing aging population with chronic and age-related diseases rapidly, resulting in subsequently increasing health care requirements in China (2), along with the increasing patients' utilization of health services (29), physicians especially in high-level hospitals on the one hand tend to have an increasingly heavier outpatient workload with worse physical health (30), and on the other hand have less service time spent with each patient averagely (31), which further contributes to inadequate communication between physicians and patients and negatively contributes to patients' perceived quality of medical services during outpatient encounters, ultimately resulting in patient dissatisfaction. When gaining insight into outpatient communication patterns, a qualitative study regarding the structure, style and focus of physician-patient communication revealed that Chinese physicians generally work alone in the outpatient clinic, and have to handle all of the work procedures by themselves during outpatient encounters, including direct interaction with their patients and paperwork (such as, recording medical history and issuing prescriptions) (32). Subsequently, our previous observational research regarding a real-time task analysis of 32 physicians in Chinese tertiary general public hospitals during outpatient encounters further revealed that during an almost 4 min outpatient encounter, a considerable amount of physician service time per patient (38.04%) was spent on non-physician-patient communication work tasks characterized by paperwork (e.g., recording medical records, and issuing prescriptions) (33). Such a high rate of physician service time was allocated to the related paperwork, which on the one hand increases the unnecessary workload (clerical burden) to physicians with occupying part of their brain resources, and on the other hand leads to less time available and brain resources for direct physician-patient interaction and further an inferior quality of medical services, ultimately resulting in physician and patient dissatisfaction (34).

When reviewing existing research on the related paperwork, there is more and more research showing that physician dissatisfaction with more time spent on paperwork and the computer but less time available for direct interaction with patients is increasing internationally (35–39) and that an increasing paperwork burden has adversely affected quality of health service delivery (40) and has become one of the important risk factors resulting in physician burnout (41, 42). Reducing the time spent on doing paperwork has become a concern to physicians as well as researchers. Hence, assessment and management of physician workload tethered to the related paperwork during outpatient encounters is of great importance to promote physical and mental well-being for physicians and decrease the risk for burnout, as well as to increase the time available for direct interaction with patients and further improve the quality of physician-patient interaction, thereby improving physician and patient satisfaction. Although current studies regarding the clerical burden of physicians have assessed the impact of adoption of electronic health records on physician workload (43–46), no previous studies have investigated the level of workload among physicians tethered to the related paperwork during outpatient encounters and its characteristics, and have assessed whether there exist distinctive workload clusters or patterns in these physicians, especially in China. Therefore, this study focused on the physician workload while performing the related paperwork during outpatient encounters.

Existing studies often simply adopted several objective workload indicators (e.g., work time, and the number of patient seen) for physician workload assessments in China, but ignored an important aspect of workload, that is, mental workload (11); and such an evaluation for physician workload is inadequate, since it cannot reflect and capture the different aspects of a physician' workload, and further explain the relation between the nature of a work task and the characteristics of the physician. Moreover, when considering paying attention to assessments of physician workload, for hospital managers, a key concern is that how to group physician workload and accurately find out individuals with high workload among the evaluated physicians to permit more targeted guidance for developing interventions to, in turn, facilitate their physical and mental health and the quality of medical services. However, internationally, there is lack of consensus on what should be considered as a threshold value for a high or excessive workload (47, 48); and current studies tend to identify individuals with high workload among the evaluated physicians by using single workload indicators (49) through the quartiles (50), or threshold values for workload [e.g., 50% of overall workload (51), >55 (12), or >60 (52) of NASA-TLX composite workload scores]. Such kind of study based on “variable-centered” methods along with human interferences on identification of physicians with high workload, although important, has obscured individual variations in the different aspects of physician workload and therefore failed to reveal the distinctive physician workload subgroups or patterns and further capture the individual characteristics associated with different physician workload groups; and thus, a “person-centered” approach may be more effective. One of the most popular and useful methods involves latent profile analysis (LPA), which provides a methodology to group individuals who share similar patterns of personal and professional characteristics, traits or behaviors into subtypes based on a set of the variables of interest (53, 54). This statistical analysis method is rather novel in the mental workload research among medical workers, and it has been shown to be usable and valid for exploring the patterns of mental workload among pandemic frontline nurses during the COVID-19 pandemic (18, 53), as well as the identification of the subtypes of physicians' mental workload in outpatient practice since the normalization of prevention and control of the COVID-19 pandemic in China (54).

There are few previous studies that further explores whether there exist distinctive workload subgroups or patterns among physicians tethered to the related paperwork during outpatient encounters. This study aimed to investigate the workload level of Chinese physicians while performing the related paperwork (such as recording medical history, and issuing prescriptions), classify the subgroups of physician workload and further examine how demographic variables differ among physicians belonging to distinctive subgroups. We hypothesized that physicians can be separated into distinctive workload subgroups based on the assessment of workload tethered to the related paperwork during outpatient encounters using the Chinese physician mental workload scale developed by our research team, and that key factors including demographic characteristics differed across distinctive subgroups. This study is the first of its subgroups of physician workload tethered to paperwork during outpatient encounters conducted in China, and can provide more targeted guidance for hospital managers to accurately find out individuals with high workload among physicians and therefore develop interventions to increase the time available and brain resources for direct interaction with patients during outpatient encounters, while lightening their paperwork burden and decreasing the risk for burnout.

## Methods

### Study Sampling and Population

This cross-sectional survey study recruited physicians in Eastern, Central, and Western China using stratified convenience sampling. To ensure sufficient representativeness, two provinces were selected in the Eastern, Central, and Western regions at the time of sampling, respectively, that is, a total of six provinces were selected. According to the standard for the division of China Eastern, Central, and Western regions from the current China Health Statistics Yearbook (7), with the consideration of the availability of sampling physicians in this survey study, Guangdong and Zhejiang provinces were selected in Eastern China, Hubei and Henan provinces were selected in Central China, and Chongqing municipality and Guangxi Zhuang autonomous region were selected in Western China. Typical sampling was then applied to select two tertiary public hospitals and two secondary public hospitals in each selected province. That is, a total of 24 public hospitals were mainly selected nationwide in China, including 12 tertiary and 12 secondary public hospitals. Among the selected hospitals, internal, surgical, obstetrics and gynecology, and pediatrics were further selected as main research departments, where targeted physicians were selected by random sampling.

Given that our survey study aimed to investigate the level of workload among Chinese physicians while performing non-physician-patient communication work tasks characterized by paperwork during outpatient encounters, the setting of the research was confined to the consulting room in outpatient clinics. Therefore, the target population was physicians who provided medical services to outpatients in outpatient clinics, those who had to have been working for at least 4 months in the outpatient clinics, and those who had to be employed full-time for at least 1 year in their current medical institution, whereas physicians who provided medical services to outpatients in outpatient clinics for <4 months, those who only provided inpatient service, and those who were graduate students or trainees were excluded in this study.

To measure the workload tethered to the related paperwork during outpatient encounters, our previous research decomposed and further divided all of the work procedures performed by physicians themselves to provide complete medical services to outpatients into the following two large groups based on a real-time task analysis of 32 Chinese physicians during outpatient encounters: “physician-patient communication work tasks” characterized by direct patient interaction, and “non-physician-patient communication work tasks” characterized by paperwork (33); and these non-physician-patient communication work tasks mainly included recording medical history, issuing medical examinations, and issuing prescriptions (33). Therefore, above-mentioned non-physician-patient communication work tasks physicians themselves performed was considered as “paperwork” during outpatient encounters in this study. Given that different types of work tasks might result in different cognitive demands and resources demands, this survey study clearly explained the detailed work tasks involved with assessed workload to the targeted physicians before they filled in the questionnaire.

### Questionnaire Design

The Chinese physician mental workload scale developed by our research team in 2018 based on the combination of dimensions of NASA-TLX scale and Subjective Workload Assessment Technology (SWAT) frameworks (11) was the basis of our developed questionnaire survey in this study, which included six dimensions (mental demands, physical demands, temporal demands, perceived risk, frustration level, and performance), 12 items, and physician characteristics (e.g., gender, age, marital status, average monthly income, educational level, professional title, working years in the current medical institution, hospital level, hospital nature, personnel, department, working hours per week, number of outpatients serviced per day, self-rated health status) with good reliability and validity (Cronbach alpha = 0.81); and moreover, pairwise comparisons of these six dimensions constituted a total of 15 comparisons, and these comparisons were used to determine the weighting coefficient for each comparison, where the weight of each dimension was equal to the number of times that dimension was selected divided by 15 (11). In the questionnaire, we added several questions to collect other demographic information on working hours per week in outpatient clinics, amount of time spent per patient and self-rated outpatient satisfaction, reported by the participating physicians.

Then, we conducted a pre-survey on site in October 2020, to validate the developed measurement tool in 10 physicians who just finished the provision of the outpatient services in the outpatient clinic of a tertiary public hospital in Wuhan, Hubei. According to their comments or feedback, context-specific adjustments were then made to improve the accuracy and clarity of the questionnaire. Because of the impact of the COVID-19 epidemic in 2020, we further used wenjuanxing, a widely-used website for conducting surveys in China, to create an electronic questionnaire with which to survey physicians in this study.

### Data Collection

This nationwide survey was conducted from November, 2020 to February, 2021. To improve the efficiency of data collection in the selected hospitals, a unique two-dimensional code of the electronic questionnaire was generated for each hospital. Prior to the beginning of the survey, an informed consent of the outpatient managers in each selected hospital was first requested and obtained, and they were then invited and volunteered to play the role of the project manager in their hospitals in this questionnaire survey. Subsequently, we sent the unique two-dimensional code of the electronic questionnaire to these outpatient managers of the corresponding hospital, and they then sent the two-dimensional code to the targeted department groups of physicians via WeChat or Tencent QQ group, where physicians who met the inclusion criteria for the targeted population were further invited to participate in this survey. Participants could scan the two-dimensional code of the electronic questionnaire via their phones to access and complete the electronic questionnaire. Before the formal survey, we introduced the purpose of the survey, provided the definition of physician workload and its involved non-physician-patient communication work tasks characterized by paperwork during outpatient encounters, and guaranteed that the survey data would not be used for other purposes. After an individual's consent was obtained, the survey was conducted accordingly. A WeChat or Tencent QQ account and mobile Internet Protocol address could be used to complete the electronic questionnaire only once. Given that the sample size should be recommended to be at least 10–15 times as many as the items of the scale (55) and should be also generally recommended to be at least 20 times as many as the variables which are considered to be included in the regression model, to improve the scale of the sample, these physicians who completed the questionnaire were also encouraged to share the survey website link to their Wechat Circle of Friends, WeChat or Tencent QQ group, where some physicians who met the inclusion criteria for the targeted population could participate in this questionnaire survey. The study was approved by the Ethics Committee of Tongji Medical College of Huazhong University of Science & Technology (No. IORG0003571).

### Workload Measure

Given that it's difficult to objectively quantify physicians' workload tethered to paperwork during outpatient encounters, we therefore used the Chinese physician mental workload scale to measure the physician workload while performing non-physician-patient communication work tasks characterized by paperwork during outpatient encounters. That is, we only used the mental workload as the measure of physician workload while performing non-physician-patient communication work tasks characterized by paperwork during outpatient encounters. The response to each of the 12 items was given based on a 10-point bipolar scale, ranging from 0 to 100; and for five of the six dimensions, i.e., mental demands, physical demands, temporal demands, perceived risk and frustration level, a score of 0 presents the lowest task load, whereas the dimension of performance is reverse-scored, with a score of 0 indicating the most successful performance of the task and the highest level of satisfaction with his/her performance (11). In this study, the calculation of physician workload followed the method from NASA-TLX scale (22); and therein the average score of all items of a corresponding dimension was the dimension score, whereas each dimension score was multiplied by the weight of the corresponding dimension and the sum of the scores was the total score of physician workload (11).

### Statistical Analysis

We performed exploratory latent profile analysis (LPA) based on the six dimension indicators of physician workload tethered to non-physician-patient work tasks characterized by paperwork during outpatient encounters in this study, where we explored homogenous subgroups in a heterogeneous group and then observed continuous variables in each subgroup. LPA, a “person-centered” statistical approach, belongs to finite mixture modeling, which can identify and describe “hidden groups” within a population (18, 54, 56). Data for the six dimension indicators of physician workload were input into the LPA, with one class initially and additional classes added incrementally, until a unique solution could not be determined; and therein the maximum parameter estimates with standard errors were applied. The model identification was checked using 200 initial stage starts and 200 final stage starts.

We tested different latent class models that categorized the physician workload patterns into one, two, three, four, five, and six groups. To determine the most appropriate latent class model, the best fit model was identified using the following key model indexes: Akaike information criterion (AIC), Bayesian information criterion (BIC), sample-size Adjusted BIC (ABIC), Lo-Mendell-Rubin (LMR), adjusted likelihood ratio test and bootstrap likelihood ratio test (BLRT) and Entropy. A lower value of AIC, BIC and ABIC represents better fitness of data into the estimated model (18, 54, 56); LMR and BLRT compare the model fit between two neighboring models (for example, k-1class model vs. k-class model), and a significant *p* value indicates that the k-class model fits the data better than the k-1-class model (54, 56). Entropy was used to assess the accuracy of classification in the estimated model, with a higher value indicating better classification, and the smallest group should have a minimum of 5% of the total sample in order to avoid over-stratification (56). A four-class model was identified in the LPA. Each participating physician was assigned into one of the physician workload subgroups with the highest probability.

Then, differences in physician workload scores among different workload subgroups were tested using the one-way analysis of variance (ANOVA) or Kruskal-Wallis rank tests. Subsequently, multinomial logistic regression analysis was performed to examine the potential relationship between the latent workload subgroups and demographic variables; and therein all demographic variables were set as independent variables since there was no collinearity problem between these demographic variables in this study, where the variance inflation factor was <10 (range: 1.07–2.65). The statistical analyses were performed using STATA (version 15.0) and Mplus (version 7.0).

## Results

### Participant Characteristics

In total, 2,038 online responses were received; of these, 104 responses were excluded because the time taken to answer the questionnaire was <60 s, or because they were not physicians, or they were physicians, but did not provide medical services to outpatients in outpatient clinics, and therefore, 1,934 eligible responses were remained. The detailed demographic characteristics of the 1,934 participating physicians are presented in Table 1. Among these physicians, 45.9% (887/1,934) were female, 44.1% (852/1,934) aged 31–40 years, 82.0% (1,585/1,934) were currently married, 63.8% (1,234/1,934) were from tertiary A hospitals, 38.0% (735/1,934) were from Eastern China, and 46.6% (902/1,934) rated health status as “moderate”. Moreover, the total mean physician workload score was 62.92 (SD = 14.70) while performing non-physician-patient communication work tasks characterized by paperwork during outpatient encounters (**Table 3**).

**Table 1:** Detailed demographic characteristics of the 1934 participating physicians.

**Characteristics**	**Number (%)**	**Characteristics**	**Number (%)**
Gender		Department	
Male	1047 (54.1)	Internal	585 (30.2)
Female	887 (45.9)	Surgical	481 (24.9)
Marital status		Obstetrics and gynecology	192 (9.9)
Unmarried	305 (15.8)	Pediatrics	163 (8.4)
Married	1585 (82.0)	Other	513 (26.5)
Divorced	36 (1.9)	Hospital level	
Widowed	8 (0.4)	Tertiary A hospital	1234 (63.8)
Age (years)		Tertiary B hospital	215 (11.1)
20–30	433 (22.4)	Secondary hospital	447 (23.1)
31–40	852 (44.1)	First-tier hospital	38 (2.0)
41–55	587 (30.4)	Working hours per week	
>55	62 (3.2)	≤40	180 (9.3)
Educational level		41–60	1062 (54.9)
PhD	228 (11.8)	>60	692 (35.8)
Postgraduate	776 (40.1)	Number of outpatients serviced per day	
Undergraduate	857 (44.3)	≤25	497 (25.7)
Junior college	59 (3.1)	26–40	582 (30.1)
Other	14 (0.7)	41–50	381 (19.7)
Professional title		>50	474 (24.5)
Senior	212 (11.0)	Outpatient working hours per week	
Deputy Senior	548 (28.3)	≤8	584 (30.2)
Intermediate	699 (36.1)	8–16	440 (22.8)
Junior	450 (23.3)	16–24	440 (22.8)
Other	25 (1.3)	24–40	268 (13.9)
Average monthly income (RMB)		>40	202 (10.4)
≤5000	376 (19.4)	Amount of time spent per patient (minutes)	
5001–10000	903 (46.7)	≤5	601 (31.1)
10001–15000	406 (21.0)	5–10	867 (44.8)
>15000	249 (12.9)	10–15	274 (14.2)
Working years in the current medical institution		>15	192 (9.9)
1–5	596 (30.8)	Self-assessed outpatient satisfaction	
6–10	503 (26.0)	Low	24 (1.2)
11–15	335 (17.3)	Medium	210 (10.9)
16–20	206 (10.7)	High	1700 (87.9)
>20	294 (15.2)	Self-assessed health status	
Area		Very poor	23 (1.2)
Eastern China	735 (38.0)	Poor	105 (5.4)
Central China	685 (35.4)	Fair	902 (46.6)
Western China	514 (26.6)	Good	624 (32.3)
Hospital nature		Very good	280 (14.5)
Public general hospital	1812 (93.7)	Personnel	
Public specialized hospital	98 (5.1)	Authorized strength	1313 (67.9)
Private general hospital	11 (0.6)	Personnel agency	201 (10.4)
Private specialized hospital	13 (0.7)	Contract	396 (20.5)
		Other	24 (1.2)

### Identification of the Subgroups of Physician Workload Tethered to Paperwork During Outpatient Encounters

In order to classify and identify the optimal model, this study extracted and compared the model solutions from the one-class to six-class models. According to model indexes, the best fitting LPA was the four-class model (Table 2), which had the lowest AIC (95,620.337), BIC (95,804.059), and ABIC (95,699.218). The *p*-values of the LMR test (<0.001) and BLRP test (<0.001) indicate that the four-class model was statistically significant. Moreover, the Entropy value (0.866 > 0.800), the proportion of physicians of the least class (8.8% > 5.0%) (Table 2) and the average profile probabilities of physicians in each category ascribed to each potential category (range: 0.920–0.930) also indicate a better classification in the four-class model.

**Table 2:** Latent profile analysis models and fit indices.

**Model**	**AIC**	**BIC**	**ABIC**	**Entropy**	**LMR *p*-value**	**BLRP *p*-value**	**Proportion of physicians in the least class**
1-class	1,02,147.568	1,02,214.376	1,02,176.252	—	—	—	—
2-class	97,880.448	97,986.227	97,925.864	0.867	<0.001	<0.001	44.2%
3-class	96,537.576	96,682.327	96599.725	0.833	0.0130	<0.001	23.8%
**4-class**	**95,620.337**	**95,804.059**	**95,699.218**	**0.866**	**<0.001**	**<0.001**	**8.8%**
5-class	95,407.784	95,630.478	95,503.397	0.813	0.1098	<0.001	5.6%
6-class	95,153.562	95,415.228	95,265.908	0.868	0.0305	<0.001	6.4%

*AIC, Akaike Information Criterion; BIC, Bayesian Information Criterion; ABIC, Sample-size-adjusted Bayesian Information Criterion; LMR, Lo-Mendell-Rubin Adjusted Likelihood Ratio Test; BLRT, Bootstrap Likelihood Ratio Test. The bold values indicate significant, and could help the readers quickly find the significant factors*.

**Figure 1:**
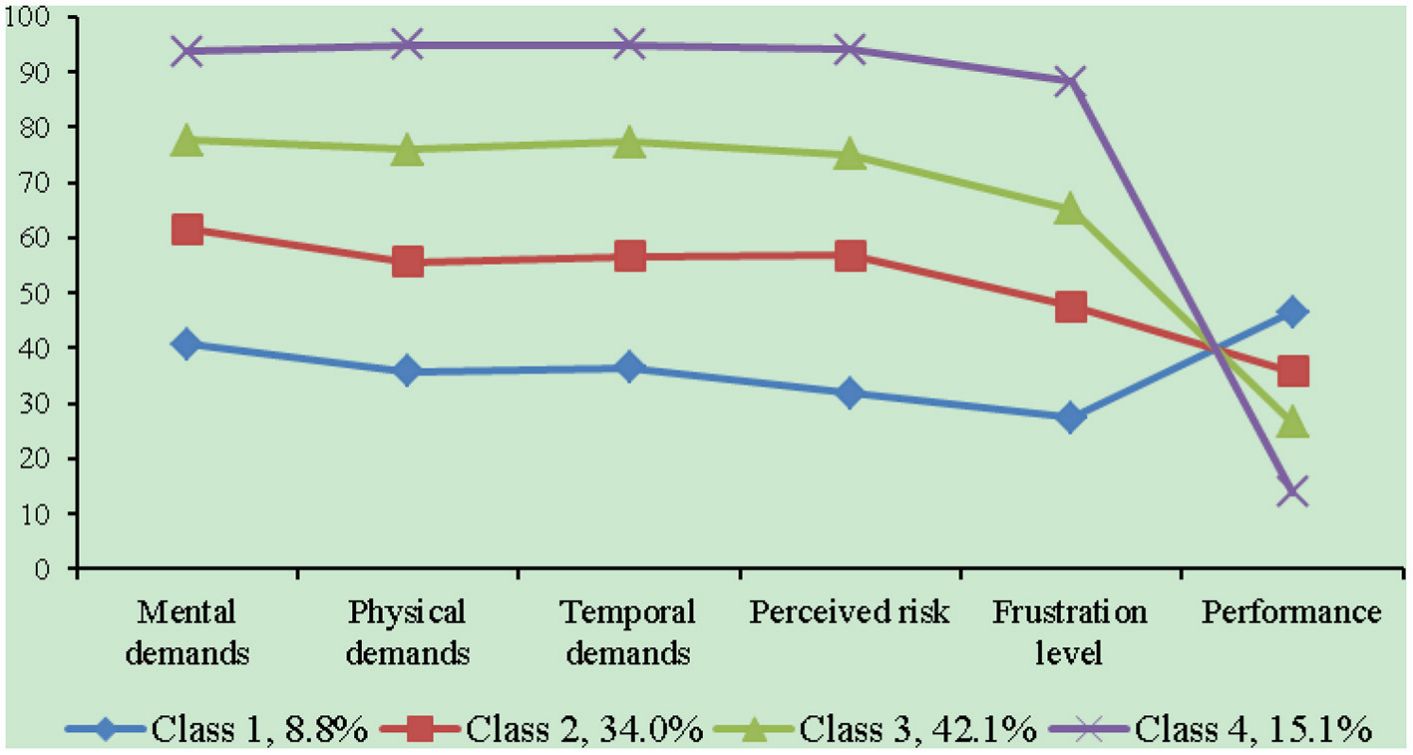
Physician workload scores in different latent classes.

Therefore, LPA identified four distinctive latent subgroups of physician workload tethered to the non-physician-patient communication work tasks characterized by paperwork during outpatient encounters. Figure 1 shows the latent subgroups of physicians (Classes 1, 2, 3, and 4), and their proportion (8.8, 34.0, 42.1, 15.1%, respectively), and the mean levels of six dimensions of physician workload, which can be distinguished as having relatively low (Class 1), medium (Class 2), high (Class 3) and very high levels (Class 4) of physician workload. That is, 8.8% (n = 170) were identified as low workload physicians (Class 1), 34.0% (n = 658) as medium workload physicians (Class 2), 42.1% (n = 814) as high workload physicians (Class 3) and 15.1% (n = 292) as very high workload physicians (Class 4). Table 3 shows comparisons of between different workload subgroups on physician workload scores, and these significant differences in total physician workload score and its dimensions scores were all found between different workload subgroups, indicating a reliable and valid grouping for physician workload tethered to the non-physician-patient communication work tasks characterized by paperwork during outpatient encounters.

**Table 3:** Comparisons of physician workload scores between different latent workload subgroups.

**Dimension indicators**	**Mean ±SD^**a**^**	**Low workload group** **(*N* = 170)**	**Medium workload group** **(*N* = 658)**	**High workload group** **(*N* = 814)**	**Very high workload group** **(*N* = 292)**	***P*** **value^b^**						
						**Low vs. Medium**	**Low vs. High**	**Low vs. Very high**	**Medium vs. High**	**Medium vs. Very high**	**High vs. Very high**	**Overall**
Mental demands	71.39 ± 18.33	40.8 ± 13.81	61.56 ± 12.61	77.72 ± 10.58	93.72 ± 8.46	<0.001	<0.001	<0.001	<0.001	<0.001	<0.001	<0.001
Physical demands	68.36 ± 19.71	35.71 ± 13.32	55.58 ± 11.64	75.98 ± 9.69	94.97 ± 6.62	<0.001	<0.001	<0.001	<0.001	<0.001	<0.001	<0.001
Temporal demands	69.37 ± 19.29	36.53 ± 13.47	56.64 ± 10.64	77.34 ± 9.08	94.97 ± 6.94	<0.001	<0.001	<0.001	<0.001	<0.001	<0.001	<0.001
Perceived risk	67.96 ± 21.00	31.82 ± 16.98	56.76 ± 14.01	75.18 ± 11.61	94.11 ± 8.05	<0.001	<0.001	<0.001	<0.001	<0.001	<0.001	<0.001
Frustration level	59.35 ± 22.82	27.41 ± 14.43	47.52 ± 15.55	65.24 ± 16.49	88.2 ± 13.68	<0.001	<0.001	<0.001	<0.001	<0.001	<0.001	<0.001
Performance	29.55 ± 17.62	46.62 ± 21.56	35.79 ± 15.55	26.55 ± 12.56	13.89 ± 16.59	<0.001	<0.001	<0.001	<0.001	<0.001	<0.001	<0.001
Total score	62.92 ± 14.70	37.55 ± 8.16	54.41 ± 6.73	68.68 ± 8.34	80.82 ± 12.08	<0.001	<0.001	<0.001	<0.001	<0.001	<0.001	<0.001

b *SD: Standard deviation*.

a *ANOVA and post-hoc pairwise Bonferroni tests for the dimensions with a normal distribution; Kruskal-Wallis equality-of-populations rank tests and post-hoc pairwise Dunn's tests for the dimensions without a normal distribution*.

Specifically, the “very high workload group” was characterized by the highest scores on the dimensions of mental demands, physical demands, temporal demands, perceived risk and frustration level, and the most successful performance of the task and the highest level of satisfaction with his/her performance, also named the “very high workload perception & very high self-evaluation group”. The “high workload group” further distinguished itself from the medium and low workload groups through higher scores on these five dimensions and more successful performance of the task and higher level of satisfaction with his/her performance, also named the “high workload perception & high self-evaluation group”, “medium workload perception & medium self-evaluation group”, and “low workload perception & low self-evaluation group”, respectively. Moreover, the gap in the physician workload scores reached 2.15 times between the very high and low workload subgroups [80.82 (SD = 12.08) vs. 37.55 (SD = 8.16), p < 0.001] (Table 3).

### Differences in the Latent Subgroups of Physician Workload by Characteristics

To further determine differences between different latent subgroups of physician workload across the demographic characteristics, multinomial logistic regression was performed to identify the significant determinants of the subgroups. Using “very high workload group” as the base outcome, we had following results (Table 4). Age, marital status, educational level, average monthly income, working years in the current medical institution, area, hospital level, hospital nature, department, working hours per week, outpatient working hours per week, number of outpatients serviced per day, amount of time spent per patient, self-assessed health status and self-assessed outpatient satisfaction were all significant factors that influenced the subgroups of physician workload tethered to non-physician-patient communication work tasks characterized by paperwork during outpatient encounters.

**Table 4:** Multinomial logistic regression results: significant determinants of latent subgroups of physician workload tethered to non-physician-patient communication work tasks characterized by paperwork during outpatient encounters (base outcome = “very high workload group”).

**Variables**	**Low workload group**		**Medium workload group**		**High workload group**	
	**RRR (95% CI)**	**p-value**	**RRR (95% CI)**	**p-value**	**RRR (95% CI)**	**p-value**
**Age (ref: 20–30 years)**						
31–40	**2.498 (1.189, 5.249)**	**0.016**	**2.061 (1.189, 3.572)**	**0.010**	**1.680 (0.988, 2.856)**	**0.056**
41–55	**2.923 (1.150, 7.429)**	**0.024**	**1.977 (0.976, 4.004)**	**0.058**	1.688 (0.858, 3.322)	0.129
>55	2.345 (0.542, 10.147)	0.254	1.223 (0.391, 3.886)	0.720	1.021 (0.339, 3.078)	0.971
**Marital status (ref: unmarried)**						
Married	**0.492 (0.244, 0.992)**	**0.047**	**0.592 (0.346, 1.014)**	**0.056**	0.801 (0.474, 1.355)	0.409
Divorced	N/A	N/A	N/A	N/A	N/A	N/A
Widowed	N/A	N/A	N/A	N/A	N/A	N/A
**Educational level (ref: PhD)**						
Postgraduate	0.711 (0.310, 1.634)	0.422	**0.458 (0.245, 0.859)**	**0.015**	0.775 (0.421, 1.431)	0.416
Undergraduate	0.604 (0.249, 1.463)	0.264	**0.386 (0.198, 0.753)**	**0.005**	**0.514 (0.268, 0.985)**	**0.045**
Junior college	2.499 (0.499, 12.502)	0.265	2.079 (0.541, 7.988)	0.286	0.858 (0.213, 3.459)	0.830
Other	N/A	N/A	N/A	N/A	N/A	N/A
**Average monthly income (ref: 10,001–15,000 RBM)**						
≤5,000	0.760 (0.351, 1.648)	0.488	**0.601 (0.337, 1.071)**	**0.084**	0.776 (0.447, 1.345)	0.366
5,001–10,000	0.712 (0.390, 1.301)	0.270	**0.639 (0.412, 0.992)**	**0.046**	**0.674 (0.442, 1.028)**	**0.067**
>15,000	1.426 (0.635, 3.202)	0.390	1.010 (0.538, 1.898)	0.974	1.290 (0.707, 2.355)	0.408
**Working years in the current medical institution (ref: 1–5 years)**						
6–10	**0.477 (0.249, 0.914)**	**0.026**	**0.583 (0.358, 0.950)**	**0.030**	0.786 (0.489, 1.265)	0.322
11–15	0.641 (0.294, 1.397)	0.263	0.688 (0.376, 1.260)	0.226	1.125 (0.627, 2.020)	0.693
16–20	**0.294 (0.117, 0.741)**	**0.009**	**0.335 (0.168, 0.668)**	**0.002**	0.632 (0.329, 1.214)	0.168
>20	0.473 (0.181, 1.235)	0.126	**0.523 (0.248, 1.104)**	**0.089**	0.838 (0.407, 1.724)	0.631
**Area (ref: central China)**						
Eastern China	**0.600 (0.353, 1.018)**	**0.058**	0.886 (0.595, 1.318)	0.549	**0.707 (0.484, 1.032)**	**0.073**
Western China	0.694 (0.399, 1.207)	0.196	0.880 (0.579, 1.338)	0.550	0.757 (0.507, 1.130)	0.173
**Hospital level (ref: tertiary B hospital)**						
Tertiary A hospital	1.445 (0.685, 3.050)	0.334	1.146 (0.659, 1.992)	0.630	**1.645 (0.962, 2.814)**	**0.069**
Secondary hospital	1.376 (0.619, 3.059)	0.433	1.604 (0.890, 2.889)	0.116	**1.810 (1.019, 3.217)**	**0.043**
First-tier hospital	0.851 (0.203, 3.568)	0.825	0.570 (0.169, 1.926)	0.365	0.571 (0.165, 1.983)	0.378
**Hospital nature (ref: public general hospital)**						
Public specialized hospital	**2.735 (1.013, 7.386)**	**0.047**	**2.031 (0.881, 4.684)**	**0.096**	1.687 (0.738, 3.857)	0.215
Private general hospital	N/A	N/A	N/A	N/A	N/A	N/A
Private specialized hospital	N/A	N/A	N/A	N/A	N/A	N/A
**Department (ref: obstetrics and gynecology)**						
Internal	1.782 (0.837, 3.794)	0.134	**2.244 (1.288, 3.910)**	**0.004**	**2.091 (1.237, 3.537)**	**0.006**
Surgical	0.994 (0.432, 2.288)	0.989	1.654 (0.901, 3.037)	0.104	**1.687 (0.948, 3.002)**	**0.075**
Pediatrics	1.558 (0.541, 4.489)	0.412	**2.974 (1.405, 6.295)**	**0.004**	**3.061 (1, 508, 6.213)**	**0.002**
Other	1.804 (0.829, 3.924)	0.137	**2.281 (1.272, 4.089)**	**0.006**	**2.288 (1.315, 3.980)**	**0.003**
**Working hours per week (ref:** **>60)**						
≤40	**6.243 (2.558, 15.240)**	**<0.001**	**5.639 (2.630, 12.091)**	**<0.001**	**3.160 (1.485, 6.722)**	**0.003**
41–60	**2.510 (1.574, 4.001)**	**<0.001**	**2.176 (1.556, 3.045)**	**<0.001**	**1.632 (1.187, 2.244)**	**0.003**
**Outpatient working hours per week (ref: 16–24)**						
≤8	1.473 (0.820, 2.648)	0.195	**1.799 (1.177, 2.750)**	**0.007**	**1.428 (0.955, 2.136)**	**0.083**
8–16	**2.001 (1.049, 3.815)**	**0.035**	**2.468 (1.543, 3.946)**	**<0.001**	**1.881 (1.204, 2.939)**	**0.006**
24–40	1.820 (0.883, 3.751)	0.105	1.268 (0.712, 2.258)	0.421	**1.650 (0.962, 2.30)**	**0.069**
>40	1.046 (0.490, 2.233)	0.908	0.944 (0.540, 1.652)	0.841	0.927 (0.548, 1.566)	0.777
**Number of outpatients serviced per day (ref: 26–40)**						
<25	0.982 (0.552, 1.746)	0.950	**1.508 (0.966, 2.355)**	**0.071**	1.074 (0.696, 1.657)	0.746
41–50	0.674 (0.371, 1.226)	0.196	0.796 (0.502, 1.263)	0.333	0.776 (0.500, 1.204)	0.258
>50	**0.421 (0.223, 0.795)**	**0.008**	**0.579 (0.362, 0.925)**	**0.022**	0.712 (0.456, 1.110)	0.134
**Amount of time spent per patient (ref: 10–15 min)**						
≤5	1.768 (0.867, 3.607)	0.117	**1.991 (1.169, 3.391)**	**0.011**	**1.812 (1.087, 3.020)**	**0.023**
5–10	**1.748 (0.924, 3.309)**	**0.086**	**1.773 (1.099, 2.860)**	**0.019**	**1.616 (1.022, 2.553)**	**0.040**
>15	1.178 (0.488, 2.841)	0.715	1.475 (0.771, 2.822)	0.241	1.613 (0.863, 3.016)	0.134
**Self-assessed health status (ref: good)**						
Very poor	N/A	N/A	N/A	N/A	N/A	N/A
Poor	0.460 (0.182, 1.163)	0.101	**0.373 (0.181, 0.766)**	**0.007**	0.679 (0.351, 1.314)	0.250
Fair	**0.487 (0.298, 0.796)**	**0.004**	**0.522 (0.359, 0.758)**	**0.001**	**0.658 (0.459, 0.942)**	**0.022**
Very good	0.703 (0.371, 1.335)	0.282	**0.577 (0.347, 0.960)**	**0.034**	**0.478 (0.290, 0.789)**	**0.004**
**Self-assessed outpatient satisfaction (ref: high)**						
Low	N/A	N/A	N/A	N/A	N/A	N/A
Fair	**3.569 (1.845, 6.904)**	**<0.001**	**2.981 (1.721, 5.164)**	**<0.001**	1.394 (0.802, 2.425)	0.239

*RRR, relative risk ratio; CI, confidence interval; ref, reference group; N/A, not applicable. The bold values indicate significant, and could help the readers quickly find the significant factors*.

Specifically, compared to those aged 20–30 years, physicians aged 31–40 years or 41–55 years were more likely to belong to the “low (RRR (Relative Risk Ratio) = 2.498, p = 0.016; RRR = 2.923, p = 0.024, respectively) or medium (RRR = 2.061, p = 0.010; RRR = 1.977, p = 0.058 <0.10, respectively) workload groups” as compared with the odds of the “very high workload group”. Physicians being married were less likely to be assigned into the “low (RRR = 0.492, p = 0.047) or medium (RRR = 0.592, p = 0.056 <0.10) workload groups”. For educational level, physicians with higher educational levels were less likely to have a higher level of workload; compared to those with a PhD degree, physicians with undergraduate degrees were less likely to belong to the “medium (RRR = 0.386, p = 0.005) or high (RRR = 0.514, p = 0.045) workload groups”, and physicians with a postgraduate degree were less likely to be assigned into the “medium workload group” (RRR = 0.458, p = 0.015) as compared with the odds of the “very high workload group”. Physicians with an average monthly income of <5,000 RMB or 5,001–10,000 RMB were less likely than those with an average monthly income of 10,001–15,000 RMB to belong to the “medium workload group” (RRR = 0.601, p = 0.084 <0.10; RRR = 0.639, p = 0.046, respectively).

Compared to those working in the current medical institution for 1–5 years, physicians who worked 6–10 years or 16–20 years in the current medical institution were less likely to belong to the “low (RRR = 0.477, p = 0.026; RRR = 0.583, p = 0.030, respectively) or medium (RRR = 0.294, P = 0.009; RRR = 0.335, p = 0.002, respectively) workload groups”. For area, physicians who were from Eastern China were less likely than those from Central China to belong to the “low (RRR = 0.600, p = 0.058 <0.10) or high (RRR = 0.707, p = 0.073 <0.10) workload groups”. Physicians in tertiary A hospitals or secondary hospitals were more likely than those in tertiary B hospitals to be assigned into the “high workload group” (RRR = 1.645, p = 0.069 <0.10; RRR = 1.810, p = 0.043, respectively). Physicians in public specialized hospitals were more likely than those in public general hospitals to belong to the “low workload group” (RRR = 2.735, p = 0.047). Moreover, compared to those in Obstetrics and Gynecology, physicians in Internal or Pediatrics were more likely to belong to the “medium (RRR = 2.244, p = 0.004; RRR = 2.974, p = 0.004, respectively) or high (RRR = 2.091, p = 0.006; RRR = 3.061, p = 0.002, respectively) workload groups” as compared with the odds of the “very high workload group”.

For working hours per week, physicians who had longer working hours per week were likely to be assigned into the “very high workload group”; compared to those with more than 60 working hours per week, physicians who worked no more than 40 h or 41–60 h were more likely to belong to the “low (RRR = 6.243, p < 0.001; RRR = 2.510, p < 0.001, respectively), medium (RRR = 5.639, p < 0.001; RRR = 2.176, p < 0.001, respectively) or high (RRR = 3.160, p = 0.003; RRR = 1.632, p = 0.003, respectively) workload groups” as compared with the odds of the “very high workload group”. Compared to those who worked 16–24 h per week in outpatient clinics, physicians with 8–16 outpatient working hours per week were more likely to be assigned in to the “low (RRR = 2.001, p = 0.035), medium (RRR = 2.468, p < 0.001), or high (RRR = 1.881, p = 0.006) workload groups”, and physicians who worked no more than 8 h in outpatient practice were more likely to belong to the “medium workload group” (RRR = 1.799, p = 0.007) as compared with the odds of the “very high workload group”.

Physicians who saw more than 50 outpatients per day were less likely than those with 26–40 outpatients serviced per day to be assigned into the “low (RRR = 0.421, *p* = 0.008) or middle (RRR = 0.579, *p* = 0.022) workload groups”. For amount of time spent per patient, the odds of belonging to the “very high workload group” increased with the time that the participating physicians spent on per patient; compared to those with 10–15 min spent per patient, physicians with no more than 5 min or 5–10 min spent per patient were more likely to be assigned into the “medium (RRR = 1.991, *p* = 0.011; RRR = 1.773, *p* = 0.019, respectively) or high (RRR = 1.813, *p* = 0.023; RRR = 1.616, *p* = 0.040, respectively) workload groups” as compared with the odds of the “very high workload group”. For self-assessed health status, physicians with worse self-assessed health status were more likely to belong to the “very high workload group”; compared to those with good health status, physicians who rated health status as “fair” were less likely to be assigned into the “low (RRR = 0.703, *p* =.004), medium (RRR = 0.522, *p* = 0.001), or high (RRR = 0.658, *p* = 0.022) workload groups”, and physicians who rated health status as poor were also less likely to be assigned into the “medium workload group” (RRR = 0.373, *p* = 0.007). Moreover, physicians who rated outpatient satisfaction as “fair” were more likely than those who rated outpatient satisfaction as “high” to belong to the “low (RRR = 3.569, *p* < 0.001) or medium (RRR = 2.981, *p* < 0.001) workload groups” as compared with the odds of the “very high workload group”.

Therefore, according to the results of multinomial logistic regression analysis, compared with the other latent workload groups, physicians belonging to the “very high workload group” were more likely to be younger, married, those who had worse health status, lower educational levels and lower average monthly incomes, those who were from Eastern China, and worked more years in the current institution, more hours per week and longer outpatient hours per week, and those who worked in public general hospitals, tertiary B hospitals and Obstetrics and Gynecology, and saw more than 50 outpatients per week with more time spent on per patient, but with high outpatient satisfaction.

## Discussion

### Principal Findings

Overall, Chinese physicians reported medium levels of workload while performing non-physician-patient communication work tasks characterized by paperwork during outpatient encounters. In this study, we identified four distinctive latent workload classes (that is, workload subgroups) among Chinese physicians: 15.1% were identified as very high workload physicians, compared with 8.8% as low workload physicians, 34.0% as medium workload physicians, and 42.1% as high workload physicians. This is a result of the combined effect of the six dimension indicators of physician workload. The “very high workload group” contributed disproportionally across all the six dimension indicators. Previous studies usually identified individuals with high workload among the evaluated physicians using single workload indicators (49) through the quartiles (50), or threshold values for workload [e.g., 50% of overall workload (51), >55 (12), or >60 (52) of NASA-TLX composite workload scores].

This study further showed that great variations in the latent workload subgroups among Chinese physicians across demographic characteristics exist. Compared with the other latent workload groups, physicians who were younger, married, those who had worse health status, lower educational levels and lower average monthly incomes, those who worked more years in the current institution, more hours per week and longer outpatient hours per week, those who worked in public general hospitals, tertiary B hospitals and Obstetrics and Gynecology, and those who saw more than 50 outpatients per day with more time spent on per patient were more likely to belong to the “very high workload group”, while performing non-physician-patient communication work tasks characterized by paperwork during outpatient encounters.

### Comparison With Prior Work

#### Level of Physician Workload Tethered to Paperwork

To the best of our knowledge, this is the first survey study to investigate the level of physician workload tethered to non-physician-patient communication work tasks characterized by paperwork during outpatient encounters and further explore its latent subgroups among Chinese physicians and identify the differences between the subgroups across demographic characteristics. Existing studies often simply adopted several objective workload indicators (e.g., work time, and the number of patient seen) for physician workload assessments in China (11), and currently, in China Health Statistics Yearbook, physicians' workloads were generally counted and measured using the average daily number of outpatients and average number of hospital beds per day that an physician undertakes (7); and none of them have examined the physician workload tethered to paperwork during outpatient encounters, whereas internationally current studies regarding the clerical burden of physicians have focused on the effect of adoption of electronic health records on physician workload (43–46).

Our study found that the total mean score of workload physicians perceived was 62.92 (SD = 14.70), and the latent workload subgroups by LPA showed that the total mean physician workload score in the “very high workload group” was 80.82 (SD = 12.08), indicating a high level of physician workload tethered to non-physician-patient work tasks characterized by paperwork during outpatient encounters, whereas lower levels of physician workload tethered to the adoption of electronic health records were reported not only in the study conducted by Pollack et al. (range 29.1–48.5) (44) but also in another study of Mazur et al. (53 ± 14/49 ± 16) (45). The possible reason for this difference might be due to that although all were related paperwork, detailed work tasks with different natures or aspects (e.g., detailed content and scopes of work tasks involved) might result in different cognitive demands and resources demands, thereby leading to different levels of workload. Another possible reason might be relevant to the fact that according to the definition of mental workload [that is, mental workload can be defined as the amount of cognitive resources used per unit time to reach the performance required by the task (57)], even if the same work task, different completion times require different levels of cognitive resources, and the shorter the time required to complete the task, the higher the mental resources required, whereas the time of access to completion of the non-physician-patient communication work tasks characterized by paperwork during outpatient encounters is rather limited (<2 min) (33), therefore resulting in a higher level of physician workload in this study. Moreover, as the survey in this study was conducted during the COVID-19 pandemic, indicating that physicians could serve fewer outpatients than normal in the outpatient clinics, the assessed results of physician workload tethered to paperwork might be lower than before the normal, thereby resulting in an underestimated difference compared to above previous studies. Therefore, hospital managers should consider paying more attention to work burden for physicians resulting from the non-physician-patient communication work tasks characterized by paperwork during outpatient encounters.

Although electronic health record is expected to improve the quality of health care, the use of electronic medical records is found to be associated with increased physician workload reported in several studies (45, 58, 59), resulting in an increased risk for burnout and less time available to spend with patients (42). With the widespread use of the electronic health records, it's generally critical that physician-patient interaction is maintained and clerical burden is minimized. Chinese physicians general work alone and handle all the related paperwork by themselves during outpatient encounters and only very senior physicians who have an assistant at their sides receive any help with these procedures (such as recording medical history using electronic health records system and issuing prescriptions on the computer) (32), and China has promoted and accelerated nationwide adoption of electronic health records in hospitals for more than a decade (60), thereby increasing the clerical burden for younger physicians in outpatient clinics, which contributed to the overall higher physician workload in this study than that reported in previous studies (44, 45), indicating a higher risk for burnout in Chinese physicians. Therefore, hospital managers should pay attention to the effect of the paperwork burden during outpatient encounters on physician burnout.

#### Identification of the Latent Subgroups of Physician Workload Tethered to Paperwork During Outpatient Encounters

In this study, four distinctive latent workload subgroups among Chinese physicians tethered to paperwork during outpatient encounters were identified through the LPA. Great variations in the overall workload score and its six dimensions scores across the four groups were revealed. The gap in the physician workload scores reached 2.15 times between the “very high and low workload groups” [80.82 (SD = 12.08) vs. 37.55 (SD = 8.16)], when significant differences in both total physician workload score and its dimensions scores were all found between different workload subgroups. These findings suggest a reliable and valid grouping for physician workload tethered to the non-physician-patient communication work tasks characterized by paperwork during outpatient encounters. However, no previous research has explored and identified the latent subgroups of physician workload tethered to paperwork during outpatient encounters, although some studies have identified the patterns or subtypes of mental workload among pandemic frontline medical workers during the COVID-19 pandemic (18, 53), as well as physicians in outpatient practice (54). A previous study on the mental workload level of physicians in outpatient practice since the normalization of prevention and control of the COVID-19 pandemic in China revealed that the latent profile analyses identified three different subtypes of physicians in their mental workload tethered to communication work tasks characterized by direct patient interaction in outpatient clinics (54). These findings suggest that different types of work tasks might lead to different latent subgroups of physicians in their workload, respectively, and therefore, we suggest that hospital managers should consider from the task level strengthening the management of physicians' workload, thereby possibly resulting in a better outcome.

Internationally, there is lack of consensus on what should be considered as a threshold value for a high or excessive workload (47, 48), and therefore, in a medical culture of outpatient clinics that provides only limited time for physician and patient interactions, how to identify and determine individuals with high workload within a specific group is still an important research topic for hospital managers to, in turn, take targeted interventions to effectively increase the time available for direct interaction with patients, therefore improving the quality of medical services. Previous research tends to identify individuals with high workload among the evaluated physicians by using single workload indicators (49) through the quartiles (50), or threshold values for workload [e.g., 50% of overall workload (51), >55 (12), or >60 (52) of NASA-TLX composite workload scores]. As noted in the Introduction, compared to such kind of study based on “variable-centered” methods with human interferences on identification of physicians with high workload (12, 49–52), LPA can provide a methodology to group individuals who share similar patterns of personal and professional characteristics, traits or behaviors into subtypes based on a set of workload indicators and further relatively distinguish workload among the different subgroups, where there is no need to set threshold values for workload for identifying individuals with high workload (18, 54). Therefore, the evaluated results by LPA can be more easily accepted by physicians as well as hospital managers, and can also help identify individuals with high workload who would otherwise be missed in single workload indicators.

The LPA analysis further indicated that 15.1% of Chinese physicians experienced the highest level of workload tethered to non-physician-patient communication work tasks characterized by paperwork during outpatient encounters in this study, whereas a higher share of physicians (33.8%) with the highest level of mental workload tethered to physician-patient communication work tasks in outpatient clinics was reported in the previous research (54). For physicians with limited resources, a higher workload tethered to non-physician-patient communication work tasks might mean that fewer both cognitive and time resources were available for physician-patient communication during outpatient encounters, ultimately resulting in further poorer quality of communication with patients, lower work performance of physicians and even adverse effects on the physician-patient relationship (34). There exist several approaches, such as physician assistants, nurses and medical scribes, to lighten the paperwork burden for physicians and increase efficiency, resulting in increased time for their interaction with patients, and improved quality of patient care, patient satisfaction and safety (36, 38, 61).

Given that China is still in a great demand for professional health workers (62), and it is therefore difficult to have sufficient human resources in a short time for assistant supports to reduce clerical burden for all physicians, how to efficiently utilize the limited human resources to improve the quality of physician-patient interactions during outpatient encounters while improving the clerical burden for physicians is of great concern to hospital managers. Our findings suggest that hospital mangers should consider these physicians belonging to the “highest workload group” as individuals who need interventions in priority to, in turn, increase the time and cognitive resources available for their interaction with patients during outpatient encounters, thereby resulting in improved quality of physician-patient communication, and a decreased risk for physician burnout, while lightening physician workload tethered to paperwork during outpatient encounters. Moreover, such a strategy should be based on the identification of the characteristics of individuals with high workload among the evaluated physicians.

#### Differences in the Latent Subgroups of Physician Workload by Characteristics

Previous studies have not yet revealed the association of demographic variables and the subgroups of physician workload tethered to paperwork during outpatient encounters (40–46). Our findings further indicated the characteristics of the different latent workload subgroups among Chinese physicians tethered to non-physician-patient communication work tasks characterized by paperwork during outpatient encounters, which can provide more targeted guidance for accurately determining individuals with high workload among the evaluated physicians, and therefore further develop targeted interventions for individual differences across physicians to increase the time and cognitive resources available for their interaction with patients while lightening physician workload tethered to paperwork during outpatient encounters. Among the four latent workload subgroups, the “very high workload group”, where physicians had relatively highest level of task load but with the most successful performance of the task, was also referred to as the “very high workload perception & very high self-evaluation group”, as these individuals tended to be younger, married, those who had worse health status, lower educational levels and lower average monthly incomes, those who worked more years in the current institution, more hours per week and longer outpatient hours per week, those who worked in public general hospitals, tertiary B hospitals and Obstetrics and Gynecology, and those who saw more than 50 outpatients per week with more time spent on per patient, but with high outpatient satisfaction. These results were partly supported by the findings of a previous study regarding relationship between physician-perceived electronic health record usability and physician workload that being married and more working hours per week were all significantly associated with higher physician workload (63), and another study regarding the relationship between clerical burden and characteristics of the electronic environment with physician burnout and professional satisfaction that more working hours per week was associated with lower physician satisfaction with clerical burden (37). Moreover, some results were also consistent with the finding of the study of Melnick et al. that being older was associated with lower physician workload (64), but inconsistent with the finding of the study of Shanafelt et al. that being older was associated with lower physician satisfaction with clerical burden (37). One possible reason for this difference might be relevant to the fact that younger physicians in China tend to have lower professional titles, thereby undertaking general outpatient services with a greater number of patients, and generally work alone in the outpatient clinic without an assistant for the related paperwork (except for the very senior ones, who have assistants) but with increasing use of electronic health records, thereby resulting in increased paperwork burden during outpatient encounters.

Our study also indicated that compared to those in tertiary A hospitals, physicians in tertiary B hospitals tended to have a higher level of workload tethered to paperwork during outpatient encounters; one possible explanation was that Chinese physicians in higher-level hospitals, although tended to undertake more outpatient visits (10), might gain more supports from assistants or more optimized outpatient doctor workstation for the related paperwork in outpatient practice, thereby optimizing their workload. Our research also found that being worse health status was associated with higher levels of workload tethered to paperwork during outpatient encounters. This is not surprising since that physicians' health is highly associated with their workload, and excessive workload contributes to poorer wellness of physicians reported in previous studies (10, 11). Thus, hospital managers should attach great importance to the impact of physician workload tethered to paperwork during outpatient encounters on their health. Previous research also revealed that increased paperwork burden has adversely affected quality of health service delivery (40) and become one of the important risk factors resulting in physician burnout (41, 42). These findings suggest that hospital managers should consider paying more attention to physicians belonging to the “very high workload group”, monitoring their workload in real time and taking measures to strengthen the management of their workload tethered to paperwork outpatient encounters to prevent and reduce the adverse effects of paperwork burden during outpatient encounters on the quality of physician-patient interactions, as well as to lighten their workload, thereby resulting in a decreased risk for burnout and achieved better job performance in outpatient practice. Furthermore, when further drawing insight into all work tasks performed by physicians to provide complete medical services to outpatients, we need further consider the level of physician workload tethered to physician-patient communication work tasks during outpatient encounters. That is, among the physicians belonging to the “very high workload group” as individuals who need interventions in this study, we need further identify and select these physicians who also have high levels of workload tethered to physician-patient communication work tasks during outpatient encounters as individuals who are intervened in priority finally. Such an outcome could more effectively decrease the risk for physician burnout and further achieve higher performance for the healthcare organizations when improving the quality of physician-patient interactions during outpatient encounters.

Moreover, when gaining insight into the impending issues the current health care system is facing in China, these existing issues, such as the hierarchical diagnosis and treatment system of China has not yet achieved effective triage of patients, whereas Chinese patients can freely choose a hospital for a visit, and still tend to go to high-level hospitals even for mild symptoms owing to their lack of confidence in the quality of health care provided in primary hospitals (10, 28), as well as the ever-increasing patient demands for health services but with lack of a proportional growth in the number of high-quality physicians, may be the root cause of unbalanced workload among physicians from different levels of hospitals, especially in high-level hospitals, where they tend to have an increasingly heavier outpatient workload. The key to balancing the workload among physicians from different levels of hospitals is to build an effective triage of Chinese patients. Therefore, policy makers should strengthen the construction of primary hospitals and improve service capabilities to, in turn, enhance patients' confidence in prioritizing the use of medical services in primary hospital and thereby promote the further development of the hierarchical diagnosis and treatment system of China to achieve effective triage of Chinese patients. Such an outcome based on the improvement of the external environment would help fundamentally ease the workload of physicians, especially in high-level hospitals.

## Limitations

This study was an early study investigating the level of physician workload tethered to non-physician-patient communication work tasks characterized by paperwork during outpatient encounters, and further identifying individuals with high workload among physicians. However, there are several limitations to be mentioned in this study. First, although stratified convenience sampling was primarily used to recruit physicians nationwide in China, because of the impact of the COVID-19 pandemic, we only employed an online questionnaire platform to collect data, and lower responsiveness was received in some selected hospitals, which may have limited the generalizability of our conclusions, and therefore, a unique two-dimensional code of the electronic questionnaire for each selected hospital was generated, and the outpatient managers in each selected hospital were invited to play the role of the project manager in their hospitals in this questionnaire survey. Second, data collection was self-reported by participating physicians via online survey, and therefore, there was no guarantee that the participating physicians filled out the questionnaire just after finishing the provision of the outpatient services in outpatient practice, which may result in a recall bias and thereby impact the generalizability of our conclusions, and therefore, we would extend our study by conducting survey on site in the future, where some variables (e.g., number of outpatients serviced per day, amount of time spent per patient, and outpatient satisfaction) could be measured by observations or by computer time spent on per patient averagely.

## Conclusion

Overall, Chinese physicians reported medium levels of workload while performing non-physician-patient communication work tasks characterized by paperwork during outpatient encounters. There exit four latent workload subgroups among physicians tethered to paperwork during outpatient encounters (named “low workload group”, “medium workload group”, “high workload group” and “very high workload group”) along with great individual variations among these subgroups. The characteristics of the latent “very high workload group” can help permit more targeted guidance for developing interventions with optimized human resource allocation to increase the time available for direct interaction with patients, thereby improving the quality of medical services and patient satisfaction during outpatient encounters, while lightening their paperwork burden and decreasing the risk for burnout. Therefore, we suggest that hospital managers should consider these physicians belonging to the highest workload group as individuals who need interventions in priority during outpatient encounters. Moreover, we also suggest that hospital managers should consider from the task level strengthening the management of physicians' workload, thereby possibly resulting in a better outcome. Furthermore, policy makers should promote the further development of the hierarchical diagnosis and treatment system of China to achieve effective triage of Chinese patients and thereby balance the workload among physicians from different levels of hospitals.

## Data Availability Statement

The datasets used and/or analyzed during the current study are available from the corresponding author on a reasonable request. Requests to access the datasets should be directed to hyh288@hotmail.com.

## Ethics Statement

Ethics approval was obtained from the Ethics Committee of Tongji Medical College of Huazhong University of Science & Technology (No. IORG0003571). All the survey data were kept confidential and anonymous.

## Author Contributions

YH designed the study, obtained funding, and participated in the collection. DL contributed to the design of this study, the acquisition, analysis, interpretation of survey data, and drafted the manuscript. SLi and CL participated in the data cleaning. SLi, CL, and YH performed revisions of the manuscript. SLi, CL, JL, and JZ contributed to the interpretation of the results. YZ, JL, JZ, and SLu were involved in data cleaning. All authors have read and approved the final version of the manuscript.

## Funding

This study was supported by the National Natural Science Foundation of China (Grant Number 71774062).

## Conflict of Interest

The authors declare that the research was conducted in the absence of any commercial or financial relationships that could be construed as a potential conflict of interest.

## Publisher's Note

All claims expressed in this article are solely those of the authors and do not necessarily represent those of their affiliated organizations, or those of the publisher, the editors and the reviewers. Any product that may be evaluated in this article, or claim that may be made by its manufacturer, is not guaranteed or endorsed by the publisher.
